# Supplementary material for: StM171, a Stenotrophomonas maltophilia Bacteriophage That Affects Sensitivity to Antibiotics in Host Bacteria and Their Biofilm Formation
Source: Viruses. 2023 Dec 18;15(12):2455. doi: 10.3390/v15122455 (PMC10747581; doi:10.3390/v15122455)
Supplement: Supplementary file 1 [file viruses-15-02455-s001.zip › Supplementary Tables/Table S1.pdf]

Table S1. List of tested *Stenotrophomonas maltophilia* and *Pseudomonas aeruginosa* strains in STM171 host range experiment

	**Strain number**	**Susceptibility to StM171 phage**	**GenBank accession**
Stenotrophomonas maltophilia			
1	2142	+	MZ424754
2	2355	+	OP393915
3	3659	+	MT040043
4	3664	+	MT040044
5	3670	+	MT040045
6	3672	-	MT040046
7	3773	-	MZ424763
8	3806	-	MZ424764
9	3963	-	MZ424756
10	4125	-	MZ424762
Pseudomonas aeruginosa			
1	1125	-	OP800156
2	1589	-	OP800159
3	1697	-	OP541579
4	1699	-	OR140575
5	1710	-	OR140576
6	1711	-	OR140576
7	1779	-	OP541582
8	2273	-	OP602241
9	2887	-	OR140577
10	2888	-	OR140577
11	3217	-	OR140578
12	3218	-	OR140578
13	3414	-	OR140579
14	3416	-	OR140580
15	3451	-	OR140581
16	3532	-	OP541585
17	3536	-	OP541586
18	3671	-	MT040050
19	3823	-	OP541588
20	3931	-	OP541589
21	3935	-	OP602243
22	3940	-	OP541590
23	3943	-	OP541591
24	3946	-	OP541592
25	3962	-	OP541593
26	4147	-	OP541595
