# Supplementary material for: StM171, a Stenotrophomonas maltophilia Bacteriophage That Affects Sensitivity to Antibiotics in Host Bacteria and Their Biofilm Formation
Source: Viruses. 2023 Dec 18;15(12):2455. doi: 10.3390/v15122455 (PMC10747581; doi:10.3390/v15122455)
Supplement: Supplementary file 1 [file viruses-15-02455-s001.zip › Supplementary Tables/Table S2.pdf]

Table S2. Antibiotics tested in the against StM171 bacterial hosts

	**Antibiotic**	**Class**	**Concentration (µg/disk)**	**CEMTC2142**	**CEMTC2355**	**CEMTC3659**	**CEMTC3664**	**CEMTC3670**
1	Gentamicin	aminoglycoside	10	susceptible	susceptible	susceptible	susceptible	susceptible
2	Amikacin	aminoglycoside	30	susceptible	susceptible	susceptible	susceptible	susceptible
3	Ampicillin	beta-lactam	10	resistant	resistant	resistant	resistant	resistant
4	Amoxicillin	beta-lactam	30	resistant	resistant	susceptible	susceptible	susceptible
5	Ampicillin-sulbactam	beta-lactamase inhibitor	20	resistant	resistant	resistant	resistant	resistant
6	Piperacillin / Tazobactam 30:10	Beta-lactam (Ureidopenicillin)	40	susceptible	susceptible	susceptible	susceptible	resistant
7	Aztreonam	beta-lactam	30	resistant	resistant	resistant	resistant	resistant
8	Imipenem	beta-lactam	10	resistant	resistant	resistant	resistant	resistant
9	Meropenem	beta-lactam	10	resistant	resistant	resistant	resistant	resistant
10	Cefoxitin	beta-lactam	30	resistant	resistant	resistant	resistant	resistant
11	Ceftazidime	beta-lactam	10	susceptible	resistant	resistant	resistant	susceptible
12	Cefepime	beta-lactam	30	susceptible	resistant	resistant	resistant	resistant
13	chloramphenicol	Phenicol	30	susceptible	susceptible	susceptible	susceptible	susceptible
14	Levofloxacin	Fluorquinolone	5	susceptible	susceptible	susceptible	susceptible	susceptible
15	Ciprofloxacin	Fluorquinolone	5	susceptible	susceptible	susceptible	susceptible	susceptible
16	Clindamycin	Lincosamide	2	resistant	resistant	resistant	resistant	resistant
17	Linezolid	oxazolidinone	10	resistant	resistant	resistant	resistant	resistant
18	Erythromycin	macrolide	15	resistant	susceptible	susceptible	susceptible	susceptible
19	Penicillin	beta-lactam	1 unit	resistant	resistant	resistant	resistant	resistant
20	Oxacillin	beta-lactam	1	resistant	resistant	resistant	resistant	resistant
21	Tetracycline	tetracycline	30	susceptible	susceptible	susceptible	susceptible	susceptible
22	Tetracycline / Clavulanic acid 75/10	tetracycline	85	susceptible	susceptible	susceptible	susceptible	susceptible
23	Trimethoprim/sulfamethoxazole	Diaminopyrimidine	25	susceptible	susceptible	susceptible	susceptible	susceptible
