# Supplementary material for: StM171, a Stenotrophomonas maltophilia Bacteriophage That Affects Sensitivity to Antibiotics in Host Bacteria and Their Biofilm Formation
Source: Viruses. 2023 Dec 18;15(12):2455. doi: 10.3390/v15122455 (PMC10747581; doi:10.3390/v15122455)
Supplement: Supplementary file 1 [file viruses-15-02455-s001.zip › Supplementary Tables/Table S3.pdf]

**Table S3.** Minimum inhibitory concentrations for antibiotics used in biofilm study

| *Stenotrophomonas maltophilia* strain | Ampicillin | Gentamicin | Levofloxacin | Tetracycline | Chloramphenicol |
|---|---|---|---|---|---|
| **CEMTC 2142** | Resistant | 20 | 5 | 15 | 7.5 |
| **CEMTC 2355** | Resistant | 20 | 5 | 15 | 7.5 |
| **CEMTC 3659** | Resistant | 20 | 5 | 15 | 7.5 |
| **CEMTC 3664** | Resistant | 20 | 5 | 15 | 7.5 |
| **CEMTC 3670** | Resistant | 20 | 5 | 15 | 7.5 |

Concentrations are given in μg/mL
