# Supplementary material for: StM171, a Stenotrophomonas maltophilia Bacteriophage That Affects Sensitivity to Antibiotics in Host Bacteria and Their Biofilm Formation
Source: Viruses. 2023 Dec 18;15(12):2455. doi: 10.3390/v15122455 (PMC10747581; doi:10.3390/v15122455)
Supplement: Supplementary file 1 [file viruses-15-02455-s001.zip › Supplementary Tables/Table S4.pdf]

Table S4. Primers used for PCR screening for StM171 genome inside bacterial hosts

**Targeted gene**	**Orientation**	**primer ID**	**Sequence**	**Location**
Tail assembly gene	Forward	Frdtail171	5′CACCTACAACCCCGCCTATC 3′	5682-5071
Tail assembly gene	Reverse	Revtail171	5′CAGGTTCTCCAGCACACAGT 3′	6275-6294
Capsid gene	Forward	Frdcap171	5′CGGCCAACTACAACCACCAT 3′	37151-37170
Capsid gene	Reverse	Revcap171	5′TTGGACTGGATGACCTCGC 3′	37640-37658
