# Supplementary figures and images for: StM171, a Stenotrophomonas maltophilia Bacteriophage That Affects Sensitivity to Antibiotics in Host Bacteria and Their Biofilm Formation
Source: Viruses. 2023 Dec 18;15(12):2455. doi: 10.3390/v15122455 (PMC10747581; doi:10.3390/v15122455)

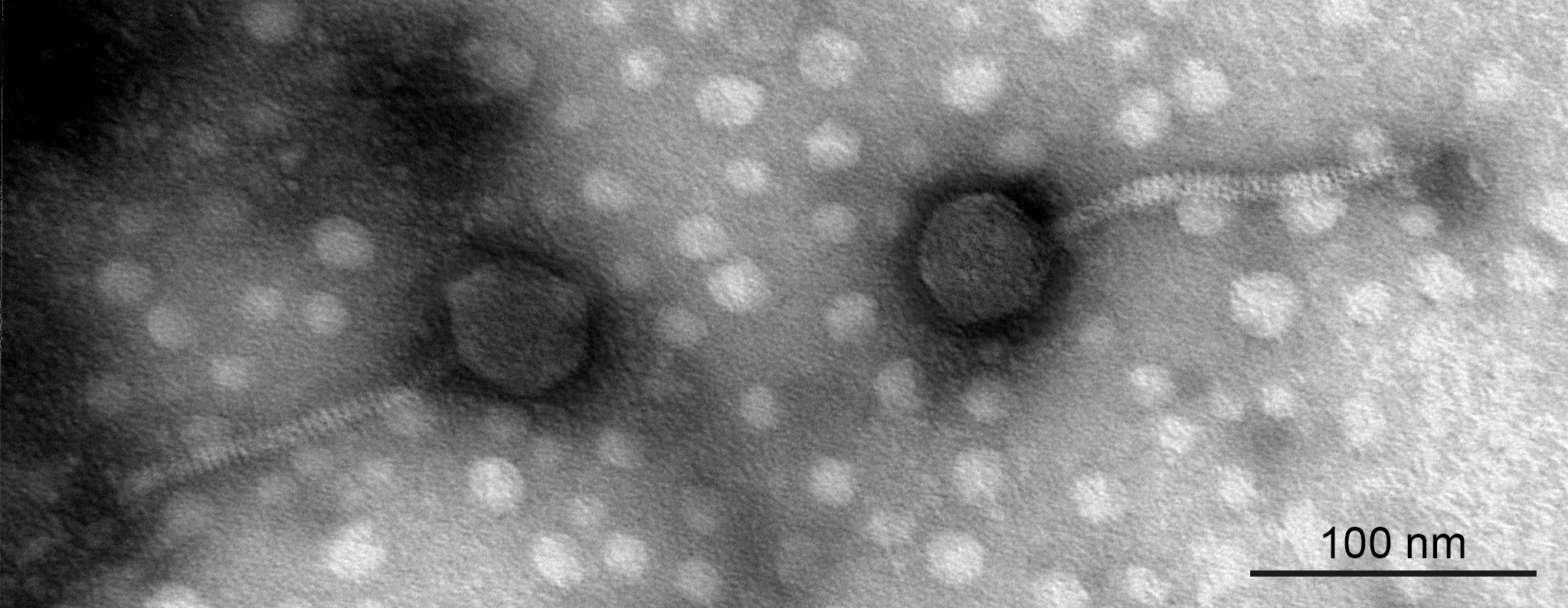

Supplement: Supplementary file 1 [file viruses-15-02455-s001.zip › Figure S1.tif]
